# Building on a Part of the Crowns of Teeth

**Published:** 1875-05

**Authors:** E. L. Hunter

**Affiliations:** Enfield, N. C.


### ARTICLE II.

### *Building on a Part of the Crowns of Teeth.*

BY E. L. HUNTER, D. D. S., ENFIELD, N. C.

It sometimes happens that, except in the pulp chamber, there is little or no retention for a filling. Take for instance a pulpless superior central incisor with the approximal, half the labial and two-thirds of the lingual walls gone. After the usual preparation, enter an inverted cone bur in the pulp chamber wall, beyond the cervical wall, and give it the shape of the bur; then take a piece of eighteen-carat gold wire, about the size of a No. 1 or 2 bur, and swell the end to correspond to the shape of the bur; bend it, so that when inserted in the pulp chamber its outer portion will intersect the point you wish occupied by the corner to be made. Now wind an unannealed cylinder on the swell end and insert wire and all into the chamber, and with smooth points and mallet condense the cylinder, holding the wire firmly in position and adding small pellets until the chamber is entirely full. It will be seen that within this chamber is the weight, on the cervical wall the fulcrum, the cutting edge the lever, and therefore that the first part of this filling must be as perfect as possible. To make this filling absolutely immovable, it is necessary to make it impossible to withdraw or move the wire where it has been planted. Such being the case, when the subsequent part of the filling has been built, the wire cannot move without crushing that part of the artificial tooth between the wire and the cervical wall. The wire should have threads cut upon it, or abrupt protuberances at several points, and be cut off at the thirty-second part of an inch short of where you wish the corner of the work. The remainder and bulk of the work, being completed with the care, and under guidance of principles usual in building, making close adaptation, will be found to have great strength.

If the case should present a long lever, that is a long tooth, with a narrow neck and wide cutting edge, it would be well to interfere with the power of leverage, by inserting a retaining screw in the body of the tooth somewhere between the cutting edge and the cervical wall.

We have used and tested this method, and we know it to be good, and offer it to those to whom necessity may never have suggested the idea.

